# Antagonizing the corticotropin releasing hormone receptor 1 with antalarmin reduces the progression of endometriosis

**DOI:** 10.1371/journal.pone.0197698

**Published:** 2018-11-14

**Authors:** Annelyn Torres-Reverón, Leslie L. Rivera-Lopez, Idhaliz Flores, Caroline B. Appleyard

**Affiliations:** 1 Dept. Neuroscience, University of Texas at Rio Grande Valley School of Medicine, Edinburg, Texas, United States of America; 2 Dept. of Human Genetics, University of Texas at Rio Grande Valley School of Medicine, Edinburg, Texas, United States of America; 3 Dept. of Psychiatry and Neurology, University of Texas at Rio Grande Valley School of Medicine, Harlingen, Texas, United States of America; 4 Division of Basic Sciences, Ponce Health Sciences University—Ponce Research Institute, Ponce, Puerto Rico; 5 Dept. of Obstetrics and Gynecology, Ponce Health Sciences University, School of Medicine, Ponce, Puerto Rico; 6 Dept. of Internal Medicine, Ponce Health Sciences University, School of Medicine, Ponce Puerto Rico; CHU Clermont-Ferrand, FRANCE

## Abstract

Endometriosis is a disorder in which endometrial tissue is found outside the uterus causing pain, infertility and stress. Finding effective, non-hormonal and long-term treatments for endometriosis still remains one of the most significant challenges in the field. Corticotropin releasing hormone (CRH) is one of the main signaling peptides within the hypothalamic pituitary adrenal (HPA) axis released in response to stress. CRH can affect nervous and visceral tissues such as the uterus and gut via activation of two types of CRH receptors: CRHR1 and CRHR2. Our aim was to determine if blocking CRHR1 with antalarmin will reduce endometriosis progression. In experiment 1 we induced endometriosis in female rats by suturing uterine horn tissue next to the intestinal mesentery and allowed to progress for 7 days. We determined that after 7 days, there was a significant increase in CRHR1 within endometriotic vesicles as compared to normal uterus. In Experiment 2, we induced endometriosis and administered either antalarmin (20 mg/kg, i.p.) or vehicle during the first 7 days after surgery. A separate group of sham surgery rats served as non-endometriosis controls. Endometriosis was allowed to progress until 60 days after surgery, at which time rats were tested for anxiety behaviors. At the time of sacrifice, endometriotic vesicles, uterus and blood were collected. Treatment with antalarmin significantly reduced the size (67% decrease) and number (30% decrease) of endometriotic vesicles. Antalarmin also prevented the increase in CRH and CRHR1 mRNA within endometriotic vesicles but not of glucocorticoid receptor. Endometriosis did not change anxiety behaviors in the open field and zero-maze tests and prior antalarmin administration did not modify this. Our data provides the first in-vivo demonstration for use of CRHR1 antagonist for the treatment of endometriosis opening the possibility for further exploring CRH signaling as a treatment target for this debilitating disease.

## Introduction

Corticotropin releasing hormone (CRH) is one of the main signaling molecules of the hypothalamic pituitary adrenal (HPA) axis. CRH has a myriad of physiological effects that include behavioral, endocrine, autonomic and immune responses [1,2]. CRH acts mainly by binding to CRH receptors type 1 (CRHR1) and type 2 (CRHR2), with a 10-fold higher affinity for the CRHR1 versus CRHR2 [3]. CRH receptors belong to the superfamily of G-protein coupled receptors and typically effect cellular activity via coupling to adenylate cyclase [3]. CRHR1 is abundant in the brain [4] as well as in adrenal glands, uterine and colonic tissues, and lymphocytes, among other tissues [5–7]. Eleven splice variants of the CRHR1 receptor have been identified [8], with a tissue-specific expression pattern [9,10]. In addition, the CRH paralog, urocortin 1 (UCN1) can bind and activate both the CRHR1 and R2 [11].

Due to the variety of physiological activities that the CRH system exerts, CRHR1 antagonists have been clinically used for more than three decades for a variety of conditions. For example, CRHR1 antagonists have been tested for the treatment of disorders including depression [12], irritable bowel syndrome (IBS) [13], and proposed as a possible treatment for anxiety disorders [14]. In fact, phase II/III clinical trials are undergoing or have been completed [15] for depression, IBS and anxiety [16]. Antalarmin is a CRHR1 antagonist, non-peptide molecule that readily crosses the blood-brain-barrier. It belongs to the family of pyrrolopyrimidine derivatives with a high affinity to CRHR1 (Ki = 0.8 nM). It has been widely used in animal research to investigate CRH effects on reproduction, inflammation, addictive disorders, and sleep disorders, among others [16, 17, 18]. Acute administration of antalarmin has been shown to block anxiety-like behaviors in response to novelty and motor activating effects of CRH [19]. In female rodents, antalarmin has been shown to prevent visceral hypersensitivity [20] suggesting that CRHR1 signaling might be a key target for understanding conditions that cause chronic pelvic pain.

Endometriosis is a chronic inflammatory disorder defined as the presence of endometrial-like tissue (e.g., glands and stroma) outside the endometrial cavity. This condition is characterized by peritoneal inflammation resulting in severe and chronic pelvic pain, and often infertility [21]. Endometriosis can be commonly misdiagnosed as IBS [22] due to overlap in common symptoms and perhaps mechanisms of disease progression involving aberrant activation of inflammatory cascades. The causes of endometriosis onset are unknown; however, a relationship between stress, hypothalamic pituitary adrenal axis (HPA) dysregulation, and endometriosis severity has been documented by others and our own work in the rat model of endometriosis [23–26]. Strong evidence (from both human and animal studies) suggest that abnormal functioning of the HPA axis, release of CRH and subsequent activation of the inflammatory response, disrupts feedback of both neuroendocrine and immune systems contributing to the development of the disease [27,28]. CRH and CRH receptors are abundant in female reproductive tissues and this axis has been shown to regulate several reproductive functions [29,30], mostly mediating pro-inflammatory activities such as ovulation, luteolysis and blastocyst implantation [2]. Despite the well-documented role of the CRH receptor in stress related disorders, reproductive function and inflammation, no previous study has addressed the potential role of CRHR1 blockade in the treatment of endometriosis.

In the current study, we took advantage of the well-established auto transplantation rat model of endometriosis to investigate the effects of the CRHR1 receptor antagonist antalarmin in endometriosis. Given the role of CRHR1 in reproductive tissues, we first tested whether this receptor was upregulated in ectopically implanted endometrium shortly after disease induction. Following this, we administered antalarmin, early during endometriosis development to test whether it could block vesicle formation in this model. We hypothesized that blockade of CRHR1 during the first week after endometriosis induction will decrease endometriosis vesicle establishment and subsequent development. In addition, we hypothesized that CRHR1 blockade may reduce stress-associated behaviors previously linked to endometriosis such as anxiety and depression. Data presented herein represent the first line of evidence for the subsequent testing of the CRHR1 antagonist antalarmin or similar compounds for reducing endometriosis.

## Materials and methods

### Animals and experimental groups

Female Sprague Dawley rats of 60 days old were used in the experiments (weighing between 190–220 grams). Rats were purchased from Ponce Research Institute Animal Facilities and littermates were never assigned to the same experimental group. Rats were housed two per cage and kept in a 12-hour light/dark cycle with food and water ad libitum. All experimental procedures were approved by the Ponce Health Sciences University (protocol #202) and the University of Texas at Rio Grande Valley (protocol #2016–004) Institutional Animal Care and Use Committees and adhere to the NIH Guide for the Care and Use of Laboratory Animals. Rats were weighed twice per week to monitor their adequate development and once a day during the drug administration period; also, estrous cycles were monitored before and after treatment to assess possible effects of the drug on reproductive cyclicity.

**Fig 1:**
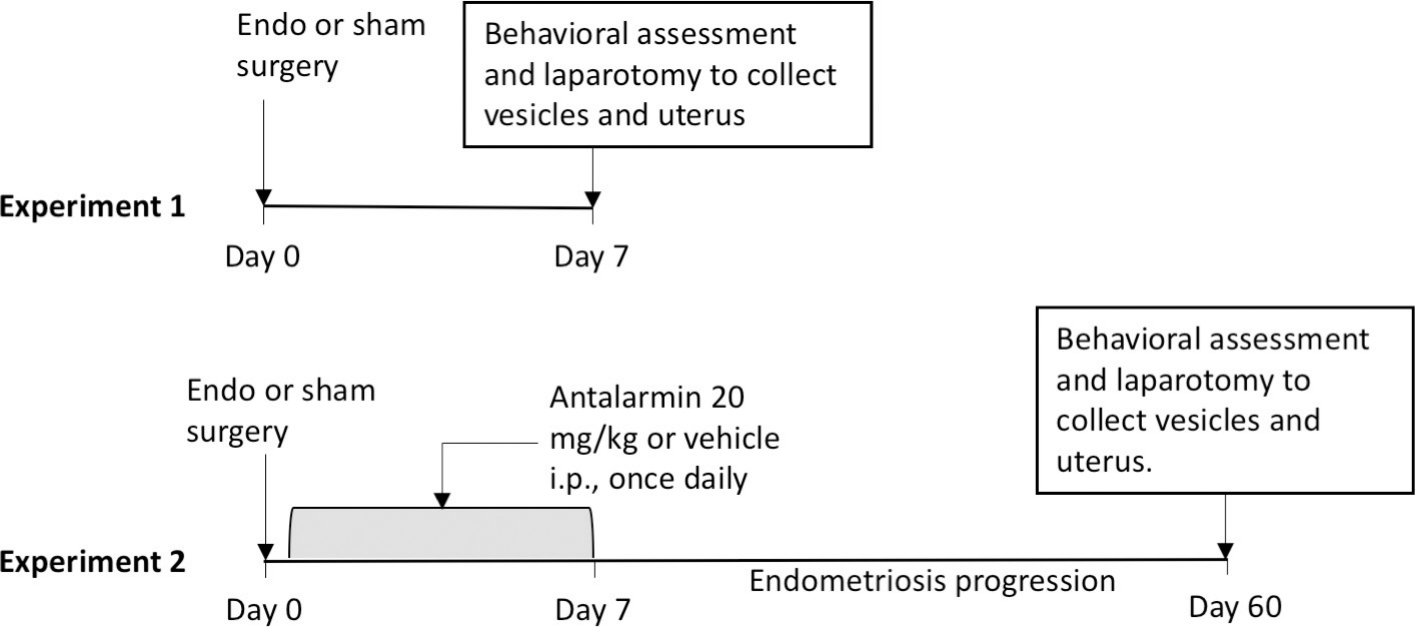
Diagram of experimental protocols. Rats in Experiment 1 received endometriosis or sham surgery and were allowed to progress for 7 days. Rats in Experiment 2 received sham surgery or endometriosis. Then the rats from the endometriosis group were injected with either vehicle (10% Tween 80) or antalarmin (i.p., once daily) for seven consecutive days after surgery and allowed to progress for 53 additional days. During the endometriosis progression period, animals were undisturbed except for weekly weighing done at the same time of cage changing.

**Experiment 1** consisted of 32 female rats that underwent endometriosis induction (n = 16) or sham surgery (n = 16; described below) and were sacrificed 7 days after surgery (surgery—Day 0; Fig 1). The main purpose of experiment 1 was to quantify the levels of CRHR1 mRNA and protein expression at 7 days after endometriosis implantation surgery and thus assess the feasibility of using a CRHR1 antagonist during this period. In addition, we collected anxiety-like behavior (open field and elevated zero maze) as well as plasma levels of corticosterone and ACTH in 8 animals from the sham group and 8 from the endometriosis group.

**Experiment 2** consisted of 51 female rats that underwent endometriosis induction (n = 40) or sham surgery (n = 11). From the 40 rats that underwent endometriosis induction, 20 rats received the CRHR1 antagonist antalarmin intraperitoneally (i.p.) and 20 rats received vehicle control i.p. (10% Tween 80) from days 1–7 after surgery and endometriosis was allowed to progress until day 60 after surgery. The remaining 11 rats that underwent sham surgery were left untreated until day 60 after surgery. The sham group was used as a non-endometriosis baseline comparison. Only rats with regular estrous cycles were used in the experiments as assessed by vaginal smear lavage during the 7 days prior to surgery and on the day of sacrifice. All but one female from the sham group showed regular estrous cycles before the surgical induction of endometriosis. Therefore, the final group numbers for endometriosis quantification and behavior were: endometriosis-antalarmin = 20, endometriosis-vehicle = 20, sham = 10. Cyclicity was not assessed during the drug administration and was only collected before treatment and the day of sacrifice. This was decided to avoid any additional stressors not related to the drug administration per se that may affect results. The estrous cycle distribution for experiments 1 and 2 can be accessed in S1 Fig.

### Endometriosis induction

Surgical induction of endometriosis followed the protocol previously published [31,32, 33]. Briefly, rats were anesthetized with isoflurane and four pieces of the right uterine horn were auto transplanted to 4 different blood vessels in the intestinal mesentery. In the sham-operated animals, sutures were placed in the intestinal mesenteric area with no uterine implants and the right uterine horn was massaged for 2 minutes. The muscle wall was closed with absorbable sutures and the skin with wound clips. Triple antibiotic was applied to the external incision after closing it. After surgery, rats were allowed to recover in individual cages until fully awake and locomotive upon which they were returned to a clean home cage with their respective cage mate. Rats were monitored for any signs of distress (piloerection, hunching, drastic changes in weight) for the 7 days following the surgery and twice weekly after that. For experiment 1, sham or endometriosis operated rats were allowed to progress for 7 days after surgery. For experiment 2, endometriosis was allowed to progress for 60 days before sacrificing, similar to our previous report [32,33].

### Drug administration in experiment 2

Animals in experiment 2 received 1 daily i.p. injection (between 09:00–10:00 hours) for 7 consecutive days of antalarmin (N-butyl-N-ethyl-[2,5,6-trimethyl-7-(2,4,6-trimethylphenyl)-7H-pyrrolo[2,3-d]pyrimidin4-yl]-amine; Tocris Bioscience, Bristol, UK) suspended in a vehicle composed of 10% Tween 80 and distilled water and given at 20 mg/kg in a volume of 1 ml/kg. This dose of antalarmin was chosen based on previous published work from Cippitelli, et al., (2012) [34]. In that dose-response study, the 20 mg/kg i.p. antalarmin administration was the most effective dose to block withdrawal behaviors. Another study also showed that antalarmin readily entered the blood-brain barrier in addition to the peripheral tissues [28]. Antalarmin injections or vehicle injections started the morning following surgery. No anesthesia was used for i.p. injections. After day 7, rats were left undisturbed except for cage change and weighing twice a week. Rats in the sham group received no injections and were weighed twice a week, similar to the other two groups.

### Behavioral assessment

One day before behavioral assessment and sacrifice a subset of rats from experiment 2 (6 vehicle and 6 antalarmin) were subjected to acute stress swim protocol of 10 min. Stressed rats were compared to no stress controls to assess how rats respond to an acute stressor. For this, animals were placed in a Plexiglass tank for 10 min in water at 25°C (modified from [35]). Rats were towel dried and kept in warm cage after swim until fur dried. The next day, we used two behavioral tasks to assess anxiety behaviors. The behavioral tests chosen take advantage of the approach-avoidance conflict that occurs in rats when presented with a novel environment. In both, the open field and the zero maze, voluntary exposure to the open, less sheltered areas is interpreted as decreased anxiety. Assessment of anxiety behaviors was carried out the same way for both experiments, experiment 1 and 2 on day 7 or 60 after surgical induction of endometriosis, respectively.

The open field test is used to quantify exploratory and locomotor activity of a rodent in an open arena. The apparatus used was a square wood arena (91 x 91 x 38 cm) with overhead light illumination and video monitoring to record animal activity using Any-Maze software (Stoelting, Wood Dale, Illinois). We quantified the following behaviors during 20 minutes: 1) total distance moved, 2) time spent moving, 3) time spent in the center of the arena, 4) time spent near the walls of the arena (defined by the 15 cm of floor arena closest to the walls) and 5) total fecal pellets. The more time the animal spends in the center of the arena compared to the space adjacent to the wall is considered as having less anxiety. At the end of the testing period, animals were returned to the home cage and after a 5-min break were tested in the elevated zero-maze. For easier comparison purposes with the zero maze (described below) and our previous publication [33], we analyzed and graphed the first 5 minutes of the open field test.

The elevated zero-maze is very similar to the more traditional elevated plus maze test, with the advantage of not having a neutral (undefined) zone in the middle. The apparatus consisted of a circle with an arm width of 10cm and elevated 40cm from floor. Two sections of the circle were enclosed by 40 cm high walls and two were open without walls. Rats were placed in the intersection of an open arm, facing the closed arm and opposite to the experimenter. Rats were recorded using the Any-Maze software (Stoelting, Wood Dale, Illinois) during 5 consecutive minutes. The following parameters were analyzed by the Any-Maze program: 1) total distance travelled in the maze, 2) time spent in the open/closed arms and 2) number of entries made by the rodent onto the open/closed arms. An entry into an arm was counted when 60% of the animal body entered it. The more time the animal spends in the open arms is considered as having less anxiety. After the 5-min testing period, the rat was returned to the home cage and immediately anesthetized with an overdose of 65% sodium pentobarbital to proceed with laparotomy. Before testing the next rat, the maze was thoroughly cleaned with 70% alcohol solution and allowed to dry.

### Sample collection and processing

We verified that the animals were deeply anesthetized. Rats were weighed, and a cytological smear taken to verify stage of the estrous cycle. The peritoneal and thoracic cavities were opened, and a blood sample was collected directly from the heart. Following this, we collected peritoneal fluid using a sterile plastic pipette. Then, we examined for the presence of endometriosis vesicles. The implants that developed into vesicles were excised from the mesentery, weighed and measured using a digital caliper. Classification of vesicles was carried out as previously described [26,36] and assigned the following grades: grade 1 = disappeared; grade 2 = 0.01–1.99 mm; grade 3 = 2–4.49 mm; grade 4 = 4.5–5.99 mm; grade 5 = 6.0 mm or larger. In sham animals, we counted and collected the empty suture sites. In addition to the endometriosis vesicles, we collected the adrenal glands, removed all surrounding fatty tissue and weighed them. We also collected blood, peritoneal fluid, colon and the left uterine horn. All tissues were flash frozen and stored at -80° until further processing.

### Enzyme linked immunosorbent assays (ELISA)

Serum and peritoneal fluid samples from animals were tested for levels of corticosterone and adrenocorticotropic hormone (ACTH). The following kits were used: Corticosterone rat/mouse kit (Cat. # 79175; IBL America, Minneapolis, MN); Mouse/rat ACTH ELISA kit (Cat. #AC018T-100, Calbiotech, El Cajon, CA).

### RNA isolation and cDNA synthesis

Normal uterine tissue and vesicles from the same animal were lysed in RLT buffer (Qiagen, Germantown, MD) and homogenized using the Bullet Blender Tissue Homogenizer (Next Advance, Averill Park, NY). RNAeasy Mini Kit (Qiagen, Germantown, MD) was used to extract the total RNA from the lysates following manufacturer's protocol. A NanoDrop 2000 UV spectrophotometer (Thermo Scientific, Wilmington, USA) was used to measure RNA concentration and purity and the quality of RNA samples was acquired based on the ratio of absorbance at 260/280 nm from the spectrophotometer. A total reaction volume of 20 μl including 1.0 μg of total RNA concentration was used to carry out the synthesis of cDNA from RNA samples using the iScript cDNA Synthesis Kit following the manufacturer’s protocol (Bio-Rad, Hercules, CA). A T-100 thermal cycler (Bio Rad, Hercules, CA) was used to carry out reactions with the following running protocol: 25°C for 5 min, 46°C for 20 min, 95°C for 1 min. Samples were stored at -80° C for later experimentation or qRT-PCR.

### Quantitative real time PCR protocol

Quantitative real time PCR (qRT-PCR) was used to evaluate changes in mRNA expression within normal uterine tissue and endometriotic vesicles. The reaction assay consisted of 25 μl of a total volume with 1:10 dilution of cDNA with IQ SyBR Green Supermix (Bio Rad Hercules, CA) in a 96 well plate. Reaction was run on a Quant Studio 12K Flex Real time PCR System (Applied Biosystems, Carlsbad, CA). Commercial primers available from Qiagen (Germantown, MD) were used for CRH, UCN1 CRHR1, CRHR2 and GR. Real time PCR cycles protocol was as follows: 95°C for 10 min. for enzyme activation followed by 40 cycles of denaturing at 95°C for 15 sec. and annealing at 60°C for 1 min. All samples were run in duplicates and normalized against GAPDH of each sample. Cycle threshold values (CT values) were automatically analyzed by the Quantstudio 12K Flex Software (Applied Biosystems, Carlsbad, CA). For standardization, the mRNA from sham rats’ uteri were always run within the same plate as the uteri and endometriotic vesicles of vehicle and antalarmin treated groups.

### Western blot in experiment 1

Western blot experiments were carried out as previously described [37] using normal uterine tissue of sham rats and the normal uterus as well as endometriosis vesicles after 7 days of endometriosis induction. We loaded 50μg of protein to identify the CRHR1 using a goat polyclonal CRHR1 antibody at a concentration of 1:250 (Abcam, Cambridge, Massachusetts, AB59023). As a loading control we used beta actin rabbit monoclonal antibody (Li-cor Biosciences, Lincoln, Nebraska, 92642210) at a concentration of 1:5000. Bands were visualized using IRDye 680 donkey anti rabbit and 800CW donkey anti goat secondary antibodies (both from Li-cor Biosciences) at a concentration of 1:15000. Membranes were imaged and analyzed using the Odyssey Clx fluorescence imaging system (Li-cor Biosciences). All lanes were quantified against the loading control beta actin.

### Statistical analyses

GraphPad Prism 7.0 (Graph-Pad Software, San Diego, California) was used to prepare graphs and run statistical analyses. Data is presented as mean difference ± SEM and a p value <0.05 was considered statistically significant. Before parametric analyses, percentages were log transformed using the formula: y = 1+[y(log)]. The variability between groups was first assessed followed by a test for outlier values. A Student t-test was used for comparisons between two groups and when group variability was significantly different, a Welch corrected t-test was used. Endometriosis vesicle measurements and behaviors were first classified within treatment group by the phase of the estrous cycle at sacrifice (proestrus, estrus, metaestrus, diestrus; see S2–S4 Figs). A Two-way analysis of variance (ANOVA) was used to first evaluate whether the estrous cycle had a significant effect or interaction with treatment. If no significant effect was present, a Student t-test or one-way ANOVA was used for between group comparisons. A one-sample t-test against the sham rats value normalized to 1.0 was used to assess qRT-PCR results. A repeated measures one-way ANOVA was used to compare changes in weight gain between treatment groups across time.

## Results

### Experiment 1

#### Behavioral parameters

**Fig 2:**
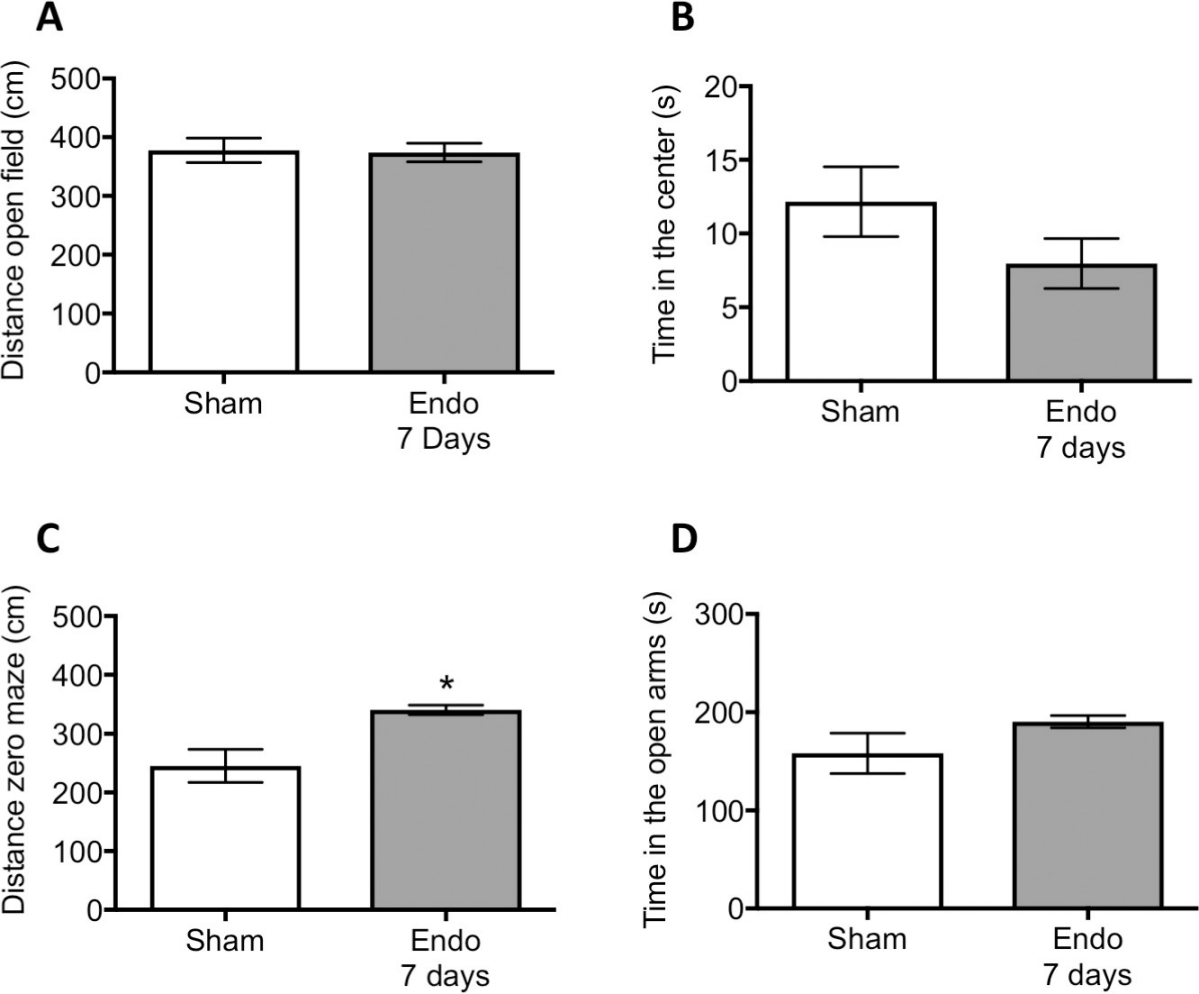
Behavioral assessment for anxiety at 7 days after sham or endometriosis surgery. (A) Total distance traveled during the first 5 minutes of the open field test and (B) time spent in the center of the arena. (C) Total distance traveled in the zero maze during the 5 min duration of the test and the amount of time spent in the open segments of the maze (D). * represents p< 0.05 compared to sham group. n = 8 in each group.

On day 7 after surgical induction of endometriosis, rats were examined for anxiety behaviors in the open field and elevated zero maze. We first analyzed the behavioral data based on the state of the estrous cycle and found no significant effects of cyclicity on the behavioral parameters. Specific behaviors by the stage of the estrous cycle can be found in S2 Fig. While all rats were recorded for 20 minutes in the open field, only the first 5 minutes are illustrated in Fig 2. Locomotion was very similar between sham and endometriosis groups. A non-significant decrease in the time in the center was observed in the rats with endometriosis at 7 days after surgery (t = 1.44, d.f. = 14, p> 0.05). Similar to the open field, no effect of estrous cycle was observed. However, a significant main effect of endometriosis induction on locomotion was observed (F_(1,12)_ = 11.26, P< 0.01; S3 Fig). Specifically, there was an increased locomotor activity at 7 days for the endometriosis group in the zero maze, compared to the sham group (Welch corrected: t = 3.24, d.f. = 8.18, p< 0.05; Fig 2). Despite increased locomotor activity in the endometriosis group, the time spent in the open arms of the arena was not significantly different from the sham group (Welch corrected: t = 1.26, d.f. = 8.83, p> 0.05). Since the number of animals at each stage of the estrous cycle at sacrifice was small, it is still unclear whether estrous cycle contributes to the initial development of endometriosis in the animal model (7 days; see S2 Fig).

#### Endometriosis development and physiological parameters

To evaluate the early endometriotic vesicle development, we induced the disease and sacrificed the animals after 7 days. We observed that 93.7% of the implants created a large vesicle, which in most cases was very large and filled with fluid. In the sham group, only sutures were observed as no endometrial tissue was transplanted. Table 1 shows the morphological characteristics of the observed vesicles at 7 days post-induction surgery.

**Table 1:** Characteristics of endometriosis vesicles at seven days after auto-transplantation surgery.

Endo vesicles at 7 days (n = 16)	Percent developed (%)	Total weight (g)	Total area (mm^2^)	Total volume (mm^3^)
Average per rat ± S.E.M.	93.75 ± 2.80	1.19 ± 0.25	160.92 ± 15.64	2190.25 ± 1110.58

**Fig 3:**
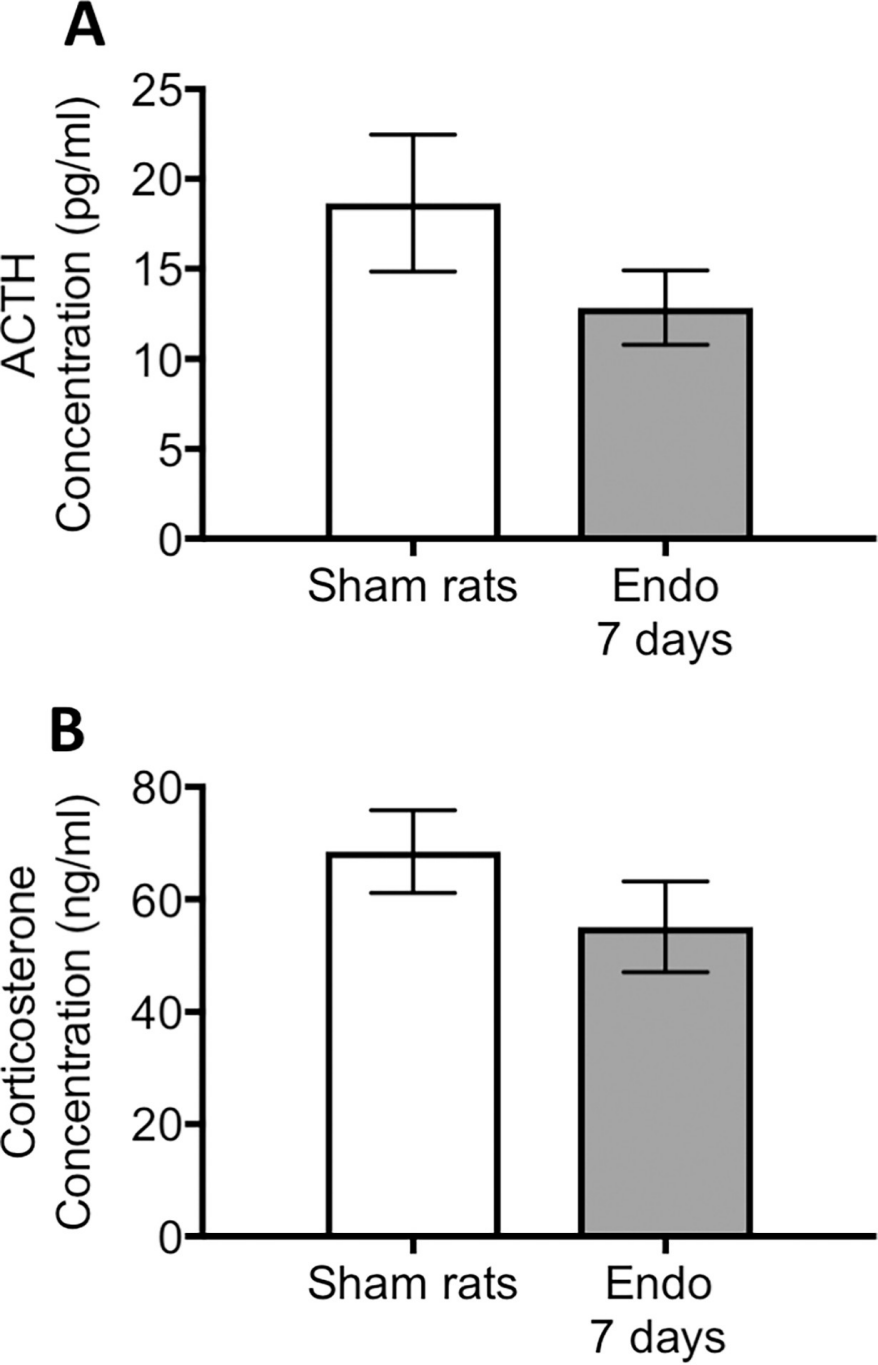
Adrenocorticotropic hormone (ACTH) and corticosterone levels in serum. (A) ACTH and (B) corticosterone were quantified in the same animals that underwent behavioral measurements (n = 8 per group). No significant differences between groups were noted for either parameter.

We and measured both, ACTH and corticosterone in serum samples from the sham and endometriosis groups. Non-significant changes in serum ACTH was noted in the group with endometriosis compared to sham group at 7 days post endometriosis induction (t = 1.34, d.f. = 14, p> 0.05; Fig 3). Corticosterone concentration in serum taken at the time of sacrifice was not different between groups either (t = 1.23, d.f. = 14, p> 0.05).

### Shortly after endometriosis induction, CRHR1 is elevated

**Fig 4:**
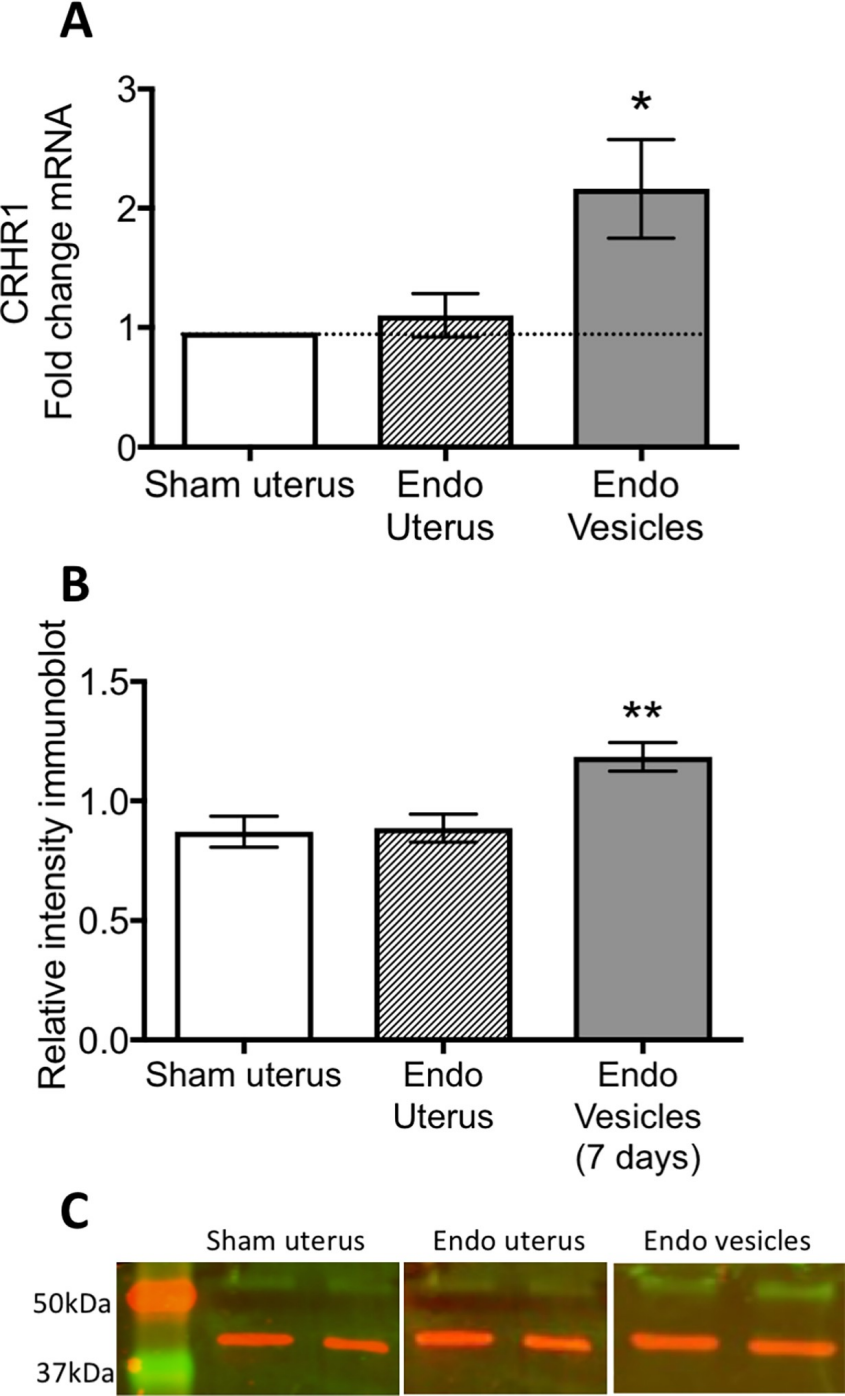
qRT-PCR and Western Blot analysis of CRHR1 within endometriosis vesicles and uterus of the endometriosis rats and sham rats. (A) At 7 days after the autotransplantation surgery to induce endometriosis, we observed a significant two-fold increase in CRHR1 mRNA within endometriosis vesicles only (n = 7 per group). (B) Western blot analysis confirmed the qRT-PCR observation showing a significant increase in CRHR1 within the endometriosis vesicles only. (C) Sample Western blot bands with the respective marker. CRHR1 was observed just above the 50 kDa marker and β-actin above the 37kDa marker. * represents p< 0.05 compared to sham group. ** represents p< 0.01 on a post-hoc analysis compared to the two other groups. Sham group n = 7, endo uterus, n = 7, endo vesicles n = 9.

We quantified the CRHR1 mRNA in the vesicles as compared to the normal uteri of the same rats and that of sham surgery controls. CRHR1 mRNA in endometriosis vesicles showed a two-fold increase as compared to normal uterus of sham rats (t = 2.934, d.f. = 6, p<0.05; Fig 4A). In contrast, the mRNA levels in uteri of rats that received endometriosis were not different from the uteri of sham rats (t = 0.829, d.f. = 6, p>0.05). To confirm the mRNA results, we followed the experiment with Western blot analysis. Protein quantification revealed a significant increase in CRHR1 within endometriosis vesicle but not within the normal uteri of the endometriosis rats (F_(2,20)_ = 8.92; p< 0.01; Fig 4B).

### Experiment 2

#### Endometriosis decreased locomotor activity which was not modified by antalarmin

To block the significant increase in CRHR1 receptor within the endometriotic vesicles, we administered antalarmin or vehicle control during the first 7 days after endometriosis induction surgery. After that, we allowed the endometriosis to progress for 53 additional days. At day 59 after endometriosis surgery, a subset of the animals (6 vehicle and 6 antalarmin) were subjected to a 5-min swim stress challenge. Antalarmin treated animals were not different from the vehicle control group in any of the behavioral parameters measured such as immobility, swimming, struggling behaviors and diving episodes (data not shown). The stress challenge did not affect the subsequent behavioral test either as all groups showed similar results (all statistics p> 0.05). Therefore, rats tested in the stress challenge were collapsed within the not-tested ones within treatment groups (vehicle or antalarmin). The next day, all animals were tested using the open field and the zero maze to evaluate trait and state anxiety, respectively. Since rats were at different stages of the estrous cycle at the time of testing, we first analyzed the behavior by cycle to verify any main effects or interaction with treatment. Estrous cyclicity at the time of behavioral testing was not a factor that explained differences in behavior for either the open field or the zero maze (S3 Fig).

**Fig 5:**
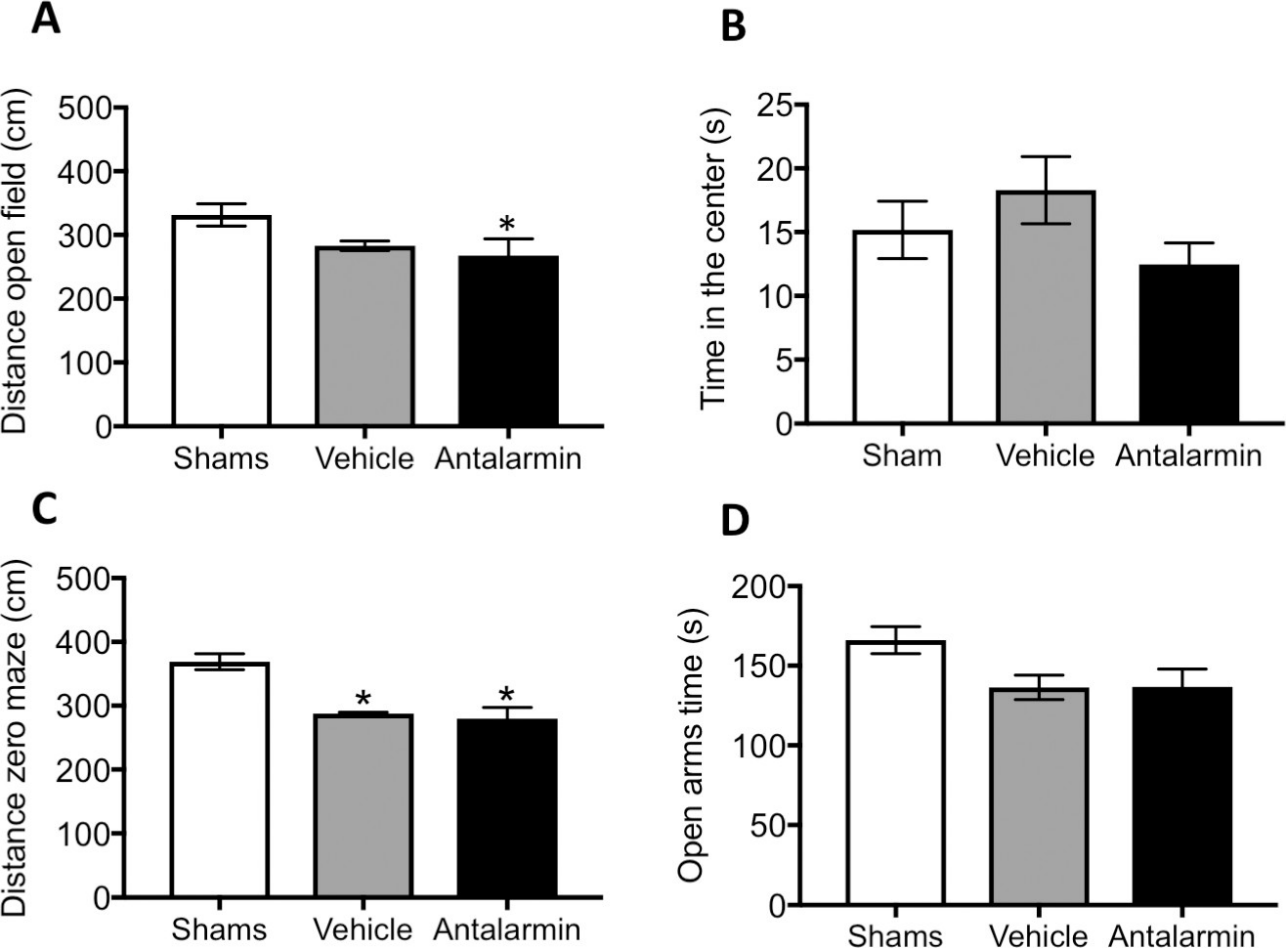
Behavioral assessment for anxiety at 60 days after sham or endometriosis surgery. Rats that received sham surgery did not receive any treatment (n = 10). Rats in the endometriosis groups received antalarmin (n = 20) or vehicle treatment (n = 20) during one week after surgery until 53 days before behavioral testing. (A and B) All animals were tested in the open field or (C and D) the elevated zero maze. (A) In comparison to sham, we observed a significant decrease in locomotor activity of the group that received antalarmin. (B) However, time spent in the center of the open field was not different between groups. (C) In the elevated zero maze, a significant decrease in locomotion was observed for rats that had endometriosis as compared to sham, regardless of the drug treatment. (D) Rats with endometriosis, regardless of drug treatment, showed a trend towards spending less time in the open segment of the zero maze as compared to sham group. * represents p< 0.05 compared to sham.

The total distance traveled during the first 5 minutes of the open field (Fig 5A) was significantly different in rats that received antalarmin as compared to the sham group (F_(2,477)_ = 3.23, p< 0.05), but the amount of time rats spend in the center of the open field arena was similar between groups (F_(2,477)_ = 0.75, p> 0.05; Fig 5B). On the zero maze, a lower locomotor activity was observed for both groups of rats with endometriosis that received vehicle or antalarmin compared to the sham group (F_(2,47)_ = 3.25, p< 0.055; post-hoc, p≤ 0.055 both comparisons; Fig 5C). Despite a lower locomotor activity, there was a strong trend for both groups of rats with endometriosis to spend less time in the open arms of the zero maze (F_(2,47)_ = 2.54, p = 0.088; Fig 5D) suggesting increase anxiety. In summary, the effects of antalarmin administered shortly after endometriosis induction produced mostly effects in locomotor activity. However, endometriosis tended to increase anxiety in the zero-maze compared to sham controls, regardless of treatment.

#### Antagonizing CRHR1 early in endometriosis produced a significant decrease in vesicle development

**Fig 6:**
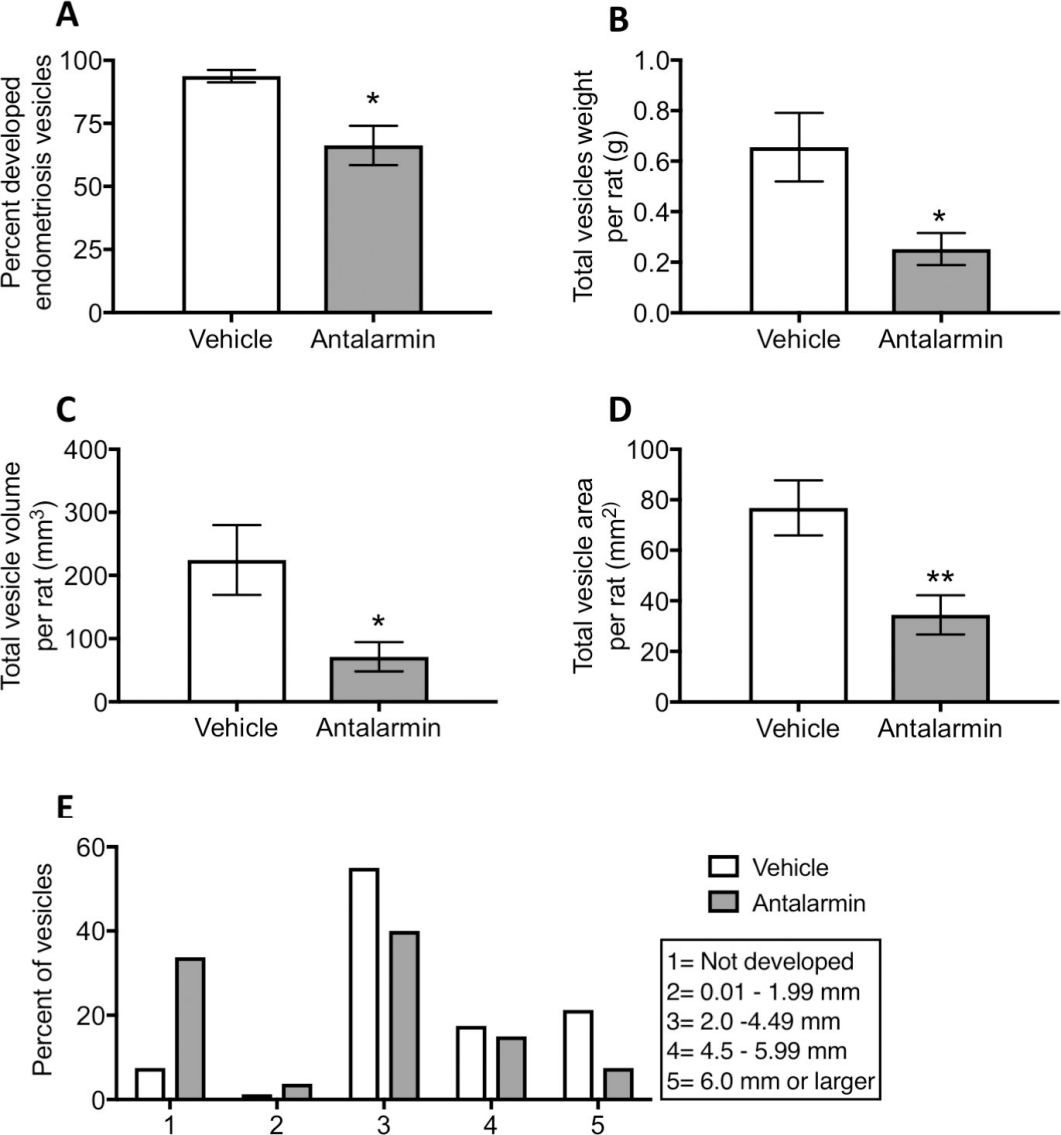
Morphological characteristics of endometriosis vesicles. (A) The percent of implants that developed into vesicles was significantly lower in the antalarmin treated group (n = 20) compared to the vehicle control group (n = 20). (B) The total weight of all vesicles per rat was smaller for the antalarmin treated rats. (C) The average vesicle volume per rat was significantly smaller for the antalarmin treated group compared to the vehicle control group. (D) The average vesicle area per rat was significantly smaller in the antalarmin group compared to the vehicle group. (E) Vesicles that developed were classified by grade based on a scale by size showing that the individual vesicles in the antalarmin treated group tended to be smaller (grade 2) or not develop at all (grade 1). * p< 0.05, ** p< 0.01.

Antalarmin administration during the 7 days after endometriosis induction resulted in a 30% significant decrease in the number of developed endometriosis vesicles at 60 days (Welch corrected t-test, t = 22.419, d.f. = 19.3, p<0.055; Fig 6A). The total weight of endometriosis vesicles (sum per rat) in the antalarmin treated group was 67% less than the vehicle control group (Welch corrected, t = 22.69, d.f. = 26.86, p = 0.01; Fig 6B). The reduced weight was a direct result of the smaller size of the vesicles in average volume (68% difference, Welch corrected, t = 2.553, d.f. = 25.45, p<0.05; Fig 6C) and area (55% difference, t = 3.16, d.f. = 38, p<0.01; Fig 6D) per rat. Similar to our previous reports, [24,32] we classified the vesicles in grades (1–5) based on a length scale for each vesicle where 1 denotes an implant that disappeared and 5 an implant that developed into a vesicle equal or larger than 6mm (Fig 6E). In the antalarmin treated group compared to the vehicle treated control, there was a larger percentage of endometriosis vesicles that disappeared, as well as a reduced percentage of vesicles of grade 3 and 5. We classified vesicle endometriosis development also by the stage of estrous cycle at the time of sacrifice. Estrous cycle did not contribute to the differences observed by treatment with antalarmin (S4 Fig). In summary, seven days of antalarmin treatment resulted in a smaller percentage of endometriosis implants developed, and those that did develop were significantly smaller in size compared to vehicle treated control group.

### Increased ACTH was observed in rats with prior antalarmin treatment

**Fig 7:**
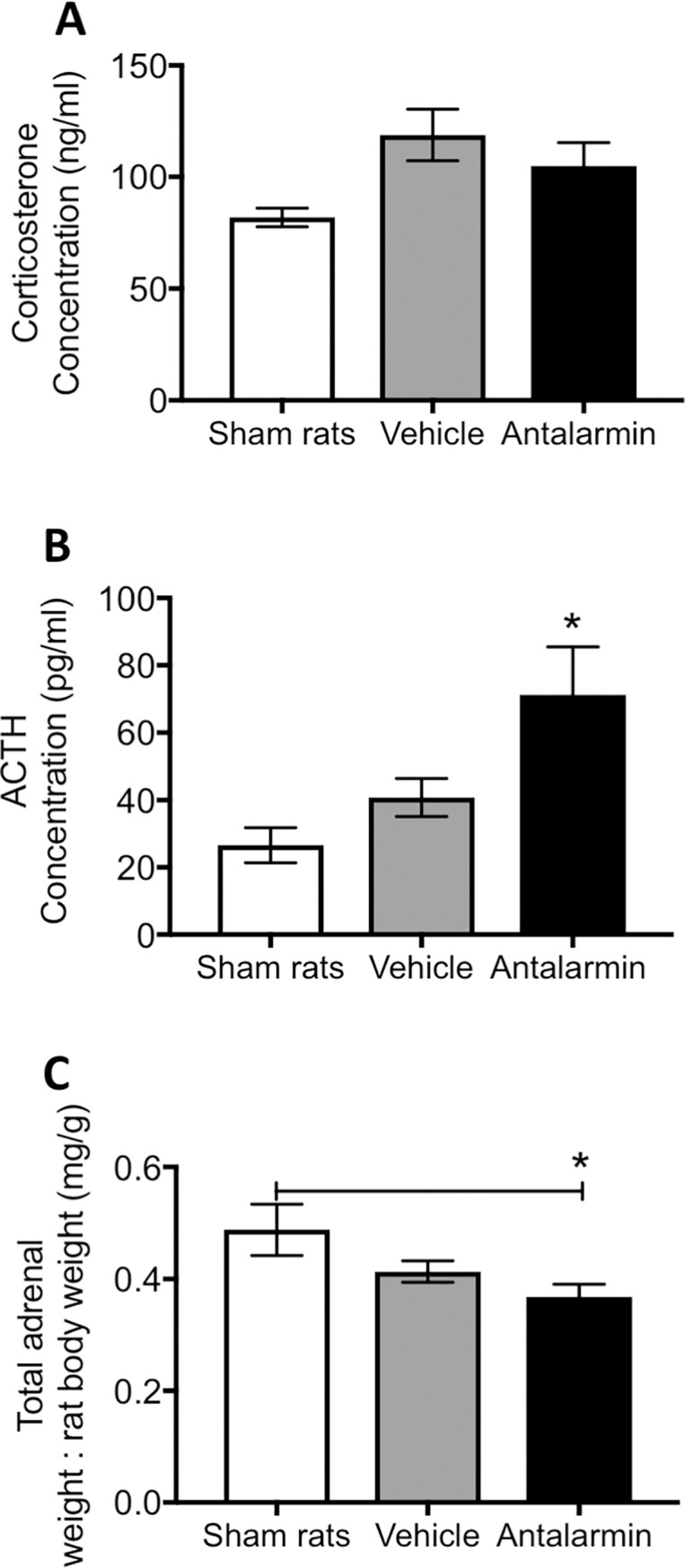
Serum corticosterone, ACTH and adrenal weight. We used ELISA to measure (A) corticosterone and (B) adrenocorticotropic hormone (ACTH) in the serum of rats. There was no significant difference between groups in serum corticosterone levels at the time of sacrifice. However, a significantly higher level of ACTH was observed for rats that received antalarmin compared to the two other groups. (C) The wet weight of adrenal glands was collected and normalized for body weight for each animal. Significantly lighter adrenal glands were noted in rats with prior antalarmin treatment compared to sham group. * represents p< 0.05.

At the time of sacrifice, we collected serum from rats to later examine corticosterone and ACTH. Corticosterone levels were slightly elevated in rats with endometriosis regardless of treatment, however this difference did not reach statistical significance (F_(2,43)_ = 1.99, p> 0.05; Fig 7A). On the other hand, we observed significantly elevated levels of serum adrenocorticotropic hormone (ACTH) in rats that received antalarmin. ANOVA statistical test revealed a significant main effect of drug treatment (F_(2,36)_ = 3.23, p = 0.05; Fig 7B). Post hoc tests showed that the groups of rats that received antalarmin was significantly higher that sham and vehicle treated groups (p< 0.05 both comparisons). In addition to the serum, we also analyzed the weight of the adrenal glands normalized to total body weight of the rat at the time of laparotomy. The wet weight of adrenals was significantly lower in rats that received antalarmin compared to the sham group (F_(2,47)_ = 4.32, p< 0.05; Fig 7C).

#### Antalarmin blocked mRNA increase in CRH and CRHR1 of uterus and vesicles

**Fig 8:**
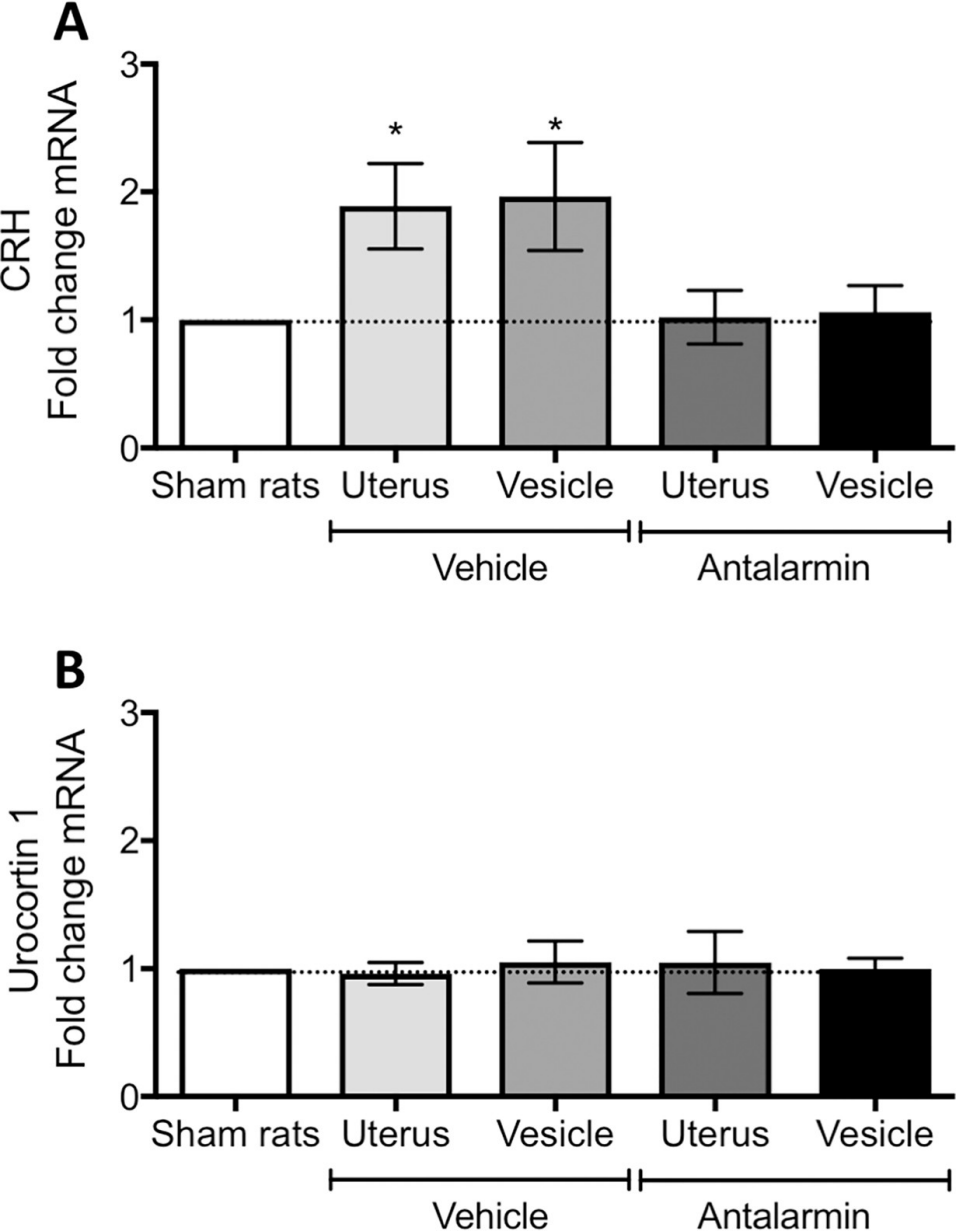
mRNA levels measured by qRT-PCR from the uterus and endometriosis vesicles. (A) corticotropin releasing hormone (CRH), n = 14 vehicle and n = 9 antalarmin. (B) Urocortin 1 peptide, n = 9 in both groups. Data normalized to the uterus of sham rats. * represents p< 0.05 compared to sham rats’ uterus.

We quantified the mRNA for urocortin and CRH, which are the main agonists of the CRHR1 receptor, within developed endometriosis vesicles in rats from both treatment groups using qRT-PCR. As a comparative parameter, we also quantified the mRNA with the uteri of the same animals and used uteri of sham controls to normalize the data. We observed a significant two-fold increase in CRH for vehicle treated rats, both in uterus (one sample t-test: t = 2.66, d.f. = 13, p< 0.05) and vesicles (t = 2.29, d.f. = 13, p< 0.05; Fig 8A). However, this increase was not observed in the antalarmin treatment group (Fig 8A). In contrast, UCN1 mRNA was not altered in any of the groups measured (Fig 8B).

**Fig 9:**
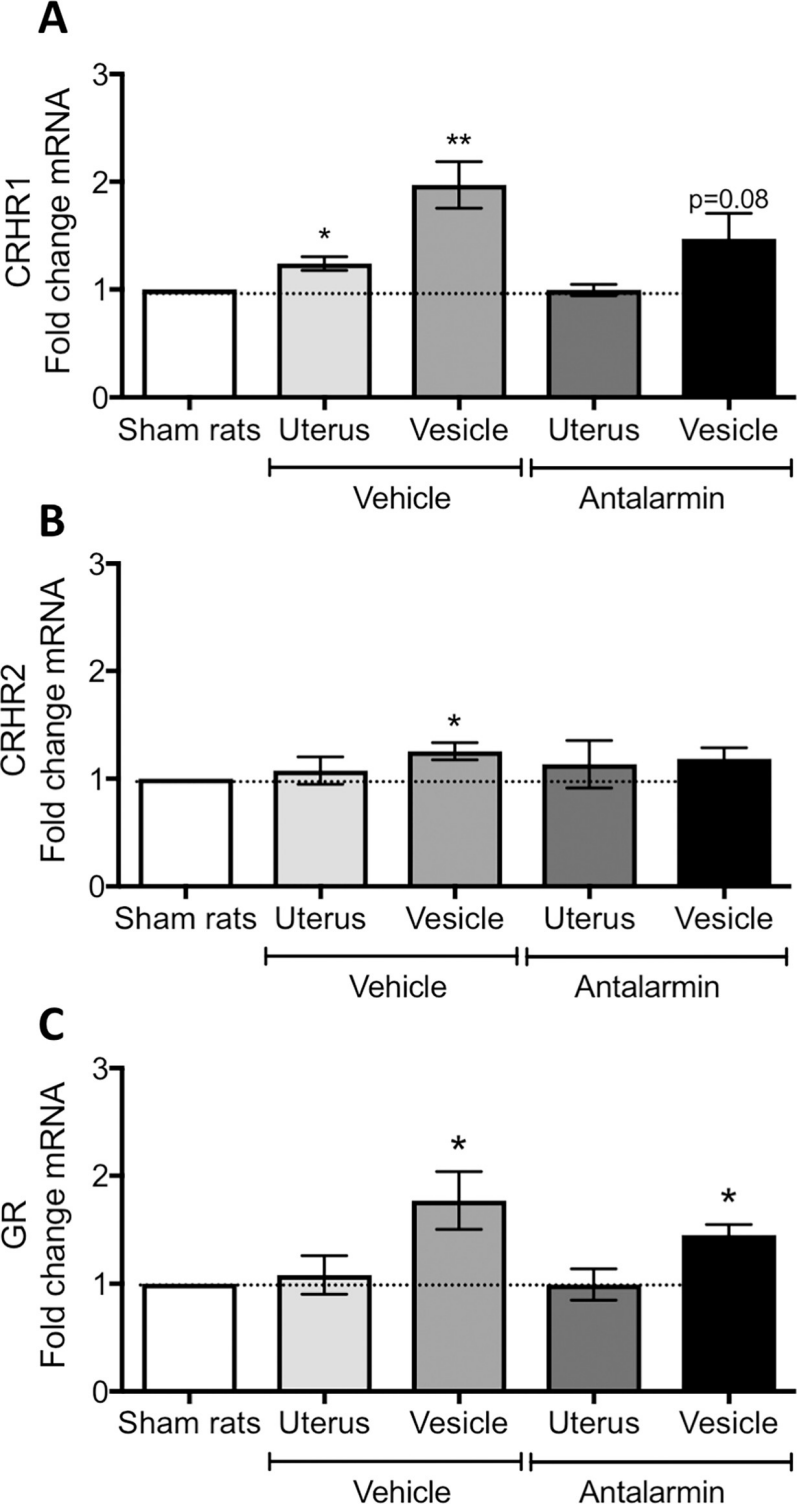
mRNA levels measured by qRT-PCR from the uterus and endometriosis vesicles. (A) corticotropin releasing hormone receptor type 1 (CRHR1). (B) corticotropin releasing hormone receptor type 2 (CRHR2). (C) Glucocorticoid receptor (GR). Data normalized to the uterus of sham rats. * represents p< 0.05 compared to sham rats’ uterus. For all panels, n = 9 in vehicle and antalarmin groups.

The mRNA of the CRHR1 receptor measured in endometriosis vesicles of the vehicle group was significantly increased as compared to sham uterus (t = 3.45, d.f. = 8, p< 0.01; Fig 9A), but this increase was not observed in the vesicles of antalarmin treated rats (p>0.05). Due to the intricate balance of CRH receptor activity in uterine tissue, we also quantified the CRHR2 receptor mRNA. For this receptor, we observed a small but significant fold increase in mRNA only in the vesicles of vehicle treated animals (t = 3.2, d.f. = 8, p< 0.05; Fig 9B). No changes were observed for CRHR2. The glucocorticoid receptor showed an interesting pattern with a significant mRNA fold increase that was observed in vesicles from both the vehicle treated (t = 2.88, d.f. = 8, p< 0.05; Fig 9C) and antalarmin treated (t = 4.65, d.f. = 8, p< 0.01; Fig 9C) groups, but no changes in uterus.

#### Antalarmin decreased body weight and the decrease persisted in treated animals

**Fig 10:**
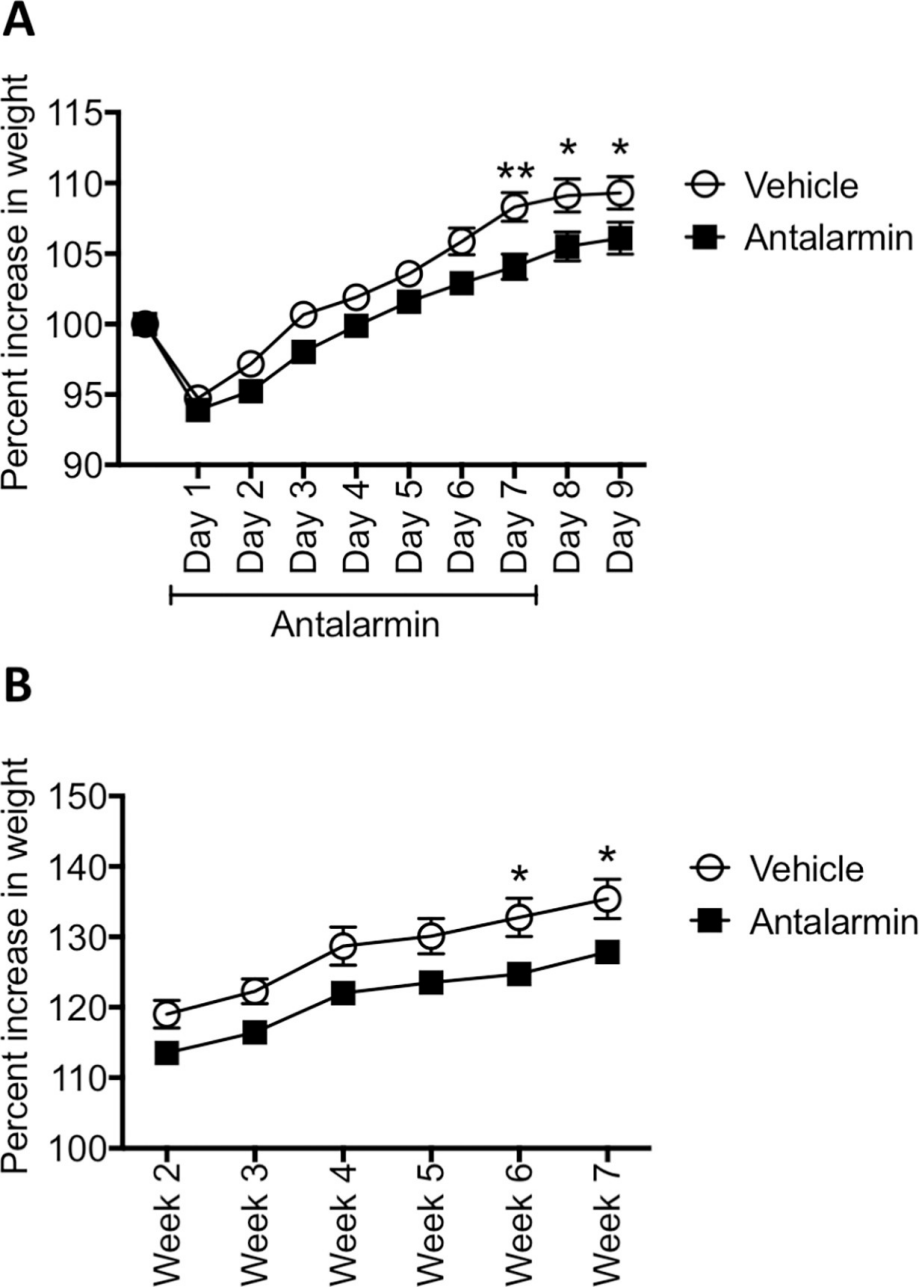
Percent increase in weight for rats with endometriosis treated with antalarmin or vehicle. (A) After seven days of treatment, antalarmin treatment significantly decreased the weight of the rats and this difference persisted for two additional days after the drug treatment has stopped. (B) During the subsequent weeks, the antalarmin group remained weighing less than control group and by weeks 6 and 7 this difference reached statistical significance. * p< 0.05, ** p< 0.01.

Inconsistencies in the effect of antalarmin on male rodent body weight have been reported [38,39]. We monitored the rat weight changes during the drug administration period and every week afterwards during the development of endometriosis. By day 7 and for 2 days after antalarmin has stopped, rats receiving the drug weighed significantly less than vehicle control groups (Repeated measures ANOVA of treatment: F_(1,38)_ = 7.615, p< 0.01, post hoc, p<0.01 on day 7 and p< 0.05 on days 8 and 9; Fig 10A). While the rats maintained a constant weight gain rate, by week 7 and 8 after endometriosis induction (Fig 10B), a significantly lower weight gain compared to vehicle group was also recorded (F_(1,38)_ = 33.89, p< 0.001, post hoc, p<0.05 for weeks 6 and 7).

## Discussion

Here we present evidence that a short treatment with the CRHR1 antagonist antalarmin was effective in reducing endometriosis with minimal changes in behavior in a rat model. Treatment reduced the number and size of vesicles that developed, suggesting that antalarmin interfered with both the development and establishment of vesicles. We also demonstrated that antalarmin prevented the increase in CRH and CRHR1 mRNA within endometriosis vesicles as compared to vehicle treated rats, and this reduction was long lasting (almost 2 months after treatment stopped). To our knowledge, our work provides the first *in vivo* evidence of efficacy and use of CRHR1 antagonist antalarmin for reducing endometriosis development using a rat model.

Treatment with antalarmin was started one day after induction of endometriosis and lasted for 7 days. At this timepoint, we documented a major increase in CRHR1 mRNA indicating a possible role of CRH signaling in the initial development of endometriotic foci. One of the major theories for endometriosis development, Sampson’s theory, postulates that during every menstruation, there is opportunity for new endometriosis implants to develop. Therefore, based on our current results, we suggest CRHR1 blockage partially impede the initial development of endometriotic sites since we still observed that about 60% of the implants sites developed into endometriosis vesicles.

It has been previously reported that treatment with antalarmin and antalarmin analogs leads to consistent decreases in anxiety-like behaviors shortly after the drug administration [19,40,41]. For these experiments, we tested antalarmin efficacy at a later time point, which would also enable comparisons to the behavioral parameters observed in our prior study using environmental enrichment [33]. Lasting changes in behavior due to prior antalarmin administration were small. This suggests that by the time of our testing, regions involved in controlling anxiety-like behaviors returned to basal conditions. While there was a decrease in locomotor activity in the endometriosis group that received antalarmin, this was not correlated with an increase in anxiety in the open field. Decreased locomotor activity in the zero maze was observed in both groups with endometriosis regardless of drug administration. These results were strikingly similar to the group with endometriosis and environmental enrichment from our previous publication [33] where we showed decreased locomotor activity in the zero maze and no effect in anxiety as measured by the time spent in the open arms. Environmental enrichment has been proven to have a direct impact on brain CRHR1 [42,43]. Therefore, mechanisms initiated via CRHR1 signaling deserve increased attention in endometriosis and might be similar to those that have been previously implicated in IBS development [20,44,45].

It is interesting that the decrease in the percentage of vesicle development in this study (30%) was very similar to our previous study that used environmental enrichment (28%; [33]). The environmental enrichment protocol that we used started before the induction of endometriosis surgery since we wanted to evaluate whether this behavioral intervention might serve as a protective factor in the progression of the disease. The similarities between the environmental manipulation and the current pharmacological manipulation, point towards a common mechanism of CRH signaling involvement in endometriosis vesicle development. One of the main differences that we observed was a hypocortisolemic state in rats with endometriosis in our previous study that was not replicated in the current study. This difference spans mainly from the different levels of corticosterone observed in the sham animals. Animals in the previous study involving environmental enrichment were brought into our behavioral facilities at postnatal day 21, weighed weekly and were handled more than the cohort of animals used in the current study. An important difference between studies is the injection protocol, which might have served as a stressor to the animals with endometriosis compared to the sham group. Future studies might take into consideration repeated sampling of corticosterone to delineate a time course of HPA axis reactivity during endometriosis progression. While it is premature to speculate on the clinical use of environmental enrichment as compared to CRHR1 antagonists for endometriosis, the fact that both studies showed a similar decrease in the vesicle development opens the possibility for exploring new treatment options targeting the CRH signaling either with pharmacology or with environmental approached aimed at reducing stress.

One of the most noteworthy findings of the current study was the elevated levels of ACTH in plasma observed 53 days after antalarmin administration has stopped. This was paralleled by a decrease in adrenal weight in animals that received antalarmin compared to both the, vehicle and sham groups. A previous report using the same dose and route of administration as in the current study showed a 31% decrease in adrenal cortex width compared to control group, but without atrophy [46]. Bornstein [47] and others [47,48] have shown decreased ACTH shortly after antalarmin was discontinued. However, to our knowledge, this is the first study looking at the effects of antalarmin several weeks after discontinuation. It is plausible that a decrease in ACTH was followed by a rebound phenomenon, largely documented during withdrawal of pharmacological agents. While further experiments are necessary to elucidate the increase in ACTH, one of the most reasonable explanations is a de-sensitization of intra-adrenal signaling system. There is evidence that human adult adrenal tissues express, ACTH, CRH, CRHR1 and CRHR2 mRNA and that exposure of adrenal cells to antalarmin blocks the production of cortisol [49]. Therefore, CRHR1 are involved in the control of glucocorticoid secretion within the adrenals and prior antalarmin administration in our model might have led to dampening or desensitization.

ACTH binds to the melanocortin receptor type 2 (MC2) to stimulate the release of glucocorticoids from the adrenal glands [50]. In human endometrium, all five types of melanocortin receptors have been found (MC1-5) and when exposed to ACTH, decreased vascularity was observed in cultured decidual biopsies [51]. In fact, ACTH has been regarded as a potent anti-inflammatory drug with proven direct activity on immune cells [52]. Therefore, we hypothesize that the observed effects of antalarmin on decreasing endometriosis progression in our current study might be indirectly mediated via ACTH.

### Limitations of the current study and future experimental approaches

Antalarmin has been reported to have anti-inflammatory activity in peripheral tissues [53,54] and specifically in endometrium [18]. While the immediate effect of antalarmin in inflammatory molecules was not the main objective of our current experiments, we recognize that inflammatory activity mainly via TNF-α, IL-6, IL-1β, among others [55,56] has a significant role in endometriosis. We do not disregard the possible contribution of immune activity in decreasing the endometriotic tissue growth as observed herein. This certainly deserves specific experimental approaches to detail the role of different cytokines and immune cells involvement following antalarmin treatment.

One of the challenges in the clinical setting is to decrease endometriosis sites, while still preserving reproductive abilities. Antalarmin has been shown in rodents to reduce the number of implantation sites by 70% [57] by a Fas-ligand immune tolerance dependent mechanism [58]. Antalarmin also produces an inhibitory effect in in vitro fertilized oocytes from mice [59]. These data in animal models suggest that antalarmin may compromise reproductive abilities in women.

The timepoint selected to administer antalarmin was based on the initial observation of mRNA and protein increase for the CRHR1 (experiment 1). However, experiments assessing the effects of antalarmin in established endometriosis are necessary. While estrous cycle stage at sacrifice was not found to influence anxiety behaviors or endometriosis vesicle size in response to antalarmin (S3 and S4 Figs), it should be noted that the number of animals in each stage of the cycle was small. Additional experimental evidence might be valuable in the near future. Along with these future experiments, evaluating if antalarmin treatment decreases pain perception would be of great benefit.

Not all CRHR1 antagonists are equal. The clinical use of CRHR1 antagonists has been limited by several factors that include lack of consistent efficacy [60,61], elevated tissue accumulation and prolonged half-life [62,63]. Recently, a group of orally administered CRHR1 antagonists have been shown to have high bioavailability and low lipophilicity in animal models of IBS [64]. The availability of these new antagonists opens significant possibilities for the advancement of testing new CRHR1 compounds in endometriosis. However, certain challenges still remain. Eleven isoforms of the CRHR1 receptor have been identified in humans [8], and splicing of CRHR1 seems to be tissue specific. For example, CRHR1β is present in pituitary myometrium and endometrium but not in adrenal, placenta or synovium [65,66]. It still needs to be determined whether ectopic endometrium will display a different profile of CRHR1 splice variants as compared to eutopic endometrium both in pre-clinical studies as well as in the clinical setting.

A recent study using intracerebroventricular administration of antalarmin showed that blocking CRHR1 provides neuroprotection and blunts neuroinflammation resulting from global cerebral ischemia [67]. Blocking CRHR1 in the hippocampus results in a reduction of excitatory activity onto CA3 pyramidal cells in hippocampus [68]. Clinically, CRHR1 signaling has been implicated in mediating abnormal brain responses to expected abdominal pain in patients with IBS [69]. Based on the significant role of CRHR1 in homeostasis and behaviors, one of the challenges will be to elucidate the role of CRHR1 peripheral blockade, compared to central blockade within specific brain structures. Additional CRHR1 antagonists that do not enter the blood brain barrier might be an effective tool to test in the endometriosis rat model.

### Conclusion

A single week of antalarmin treatment, corresponding to an up regulation of CRHR1 within endometriotic vesicles, was effective in reducing endometriosis in the rat model by reducing the number of developed vesicles by 30% and the size of the vesicles that developed by 67%. CRHR1 Inhibitors are pharmacological agents that are advanced in the pipeline of clinical trials in safety and efficacy profiles for other inflammatory disorders such as IBS. Our study opens the possibility for additional pre-clinical testing of CRHR1 inhibitors in endometriosis with the eventual translation of our work into the clinical application. It is our ultimate goal to produce novel lines of treatment that can significantly benefit many women that suffer from endometriosis.

## Supporting information

S1 Fig Estrous cycle distribution for experiment 1 and experiment 2. (A) The number of rats that were at each stage of the estrous cycle the day of behavioral testing and sacrifice for experiment 1. Estrous cycle before endometriosis surgery was not assessed in these rats. (B) Estrous cycle in rats for experiment 2 was assessed for 10 days prior to induction surgery. The percent of time that each experimental group spent in each of the phases of the estrous cycle. (C) The number of rats that were on each phase of the estrous cycle on the day of behavioral testing and sacrifice, 60 days after the induction surgery. Note that one rat in the sham group had irregular cycles and was removed from the group.
(TIF)

S2 Fig Anxiety behaviors at 7 days after endometriosis induction surgery for experiment 1. Estrous cycle stage at the time of sacrifice was measured by vaginal smear. We did not pre-planned to sacrifice at any particular stage of the cycle but rather at exactly 7 days after the endometriosis induction. (A and B) Total distance traveled in the open field and time spent in the center. (C and D) Total distance traveled in the zero maze and time spent in the open arms of the maze. Parametric analyses were not performed due to the small number of animals in each estrous cycle group. Numbers at the bottom of bars in panel A represent the number of animals per group per stage of estrous cycle. Bars represent mean ± S.E.M. in this and all subsequent supplemental figures.
(TIF)

S3 Fig Anxiety behaviors at 60 days after endometriosis induction or sham surgery for experiment 2. Similar to experiment 1, estrous cycle stage at the time of sacrifice was measured by vaginal smear. We did not pre-planned to sacrifice at any particular stage of the cycle but rather at 60 days after the endometriosis induction. (A and B) Total distance traveled in the open field and time spent in the center. (C and D) Total distance traveled in the zero maze and time spent in the open arms of the maze. The table illustrates the statistical results of the Two-way ANOVAs for each panel showing no effects of estrous cycle. Numbers at the bottom of bats in panel A represent the number of animals per group per stage of estrous cycle. It should be noted that the number of animals per group in the diestrus stage of the estrous cycle was small.
(TIF)

S4 Fig Morphological characteristics of endometriosis vesicles illustrated by the stage of estrous cycle at sacrifice. (A) Percent developed vesicles. (B) Group means of the total vesicle weight per rat. (C) Group means for the total vesicle volume per rat. (D) Group means for the total vesicle area per rat. The table illustrates the statistical results of the Two-way ANOVAs for each panel showing no effects of estrous cycle. No interactions of estrous cycle and treatment were observed. Numbers at the bottom of bars in panel A represent the number of animals per group, per stage of estrous cycle.
(TIF)
