# Supplementary figures and images for: Antagonizing the corticotropin releasing hormone receptor 1 with antalarmin reduces the progression of endometriosis
Source: PLoS One. 2018 Nov 14;13(11):e0197698. doi: 10.1371/journal.pone.0197698 (PMC6235236; doi:10.1371/journal.pone.0197698)

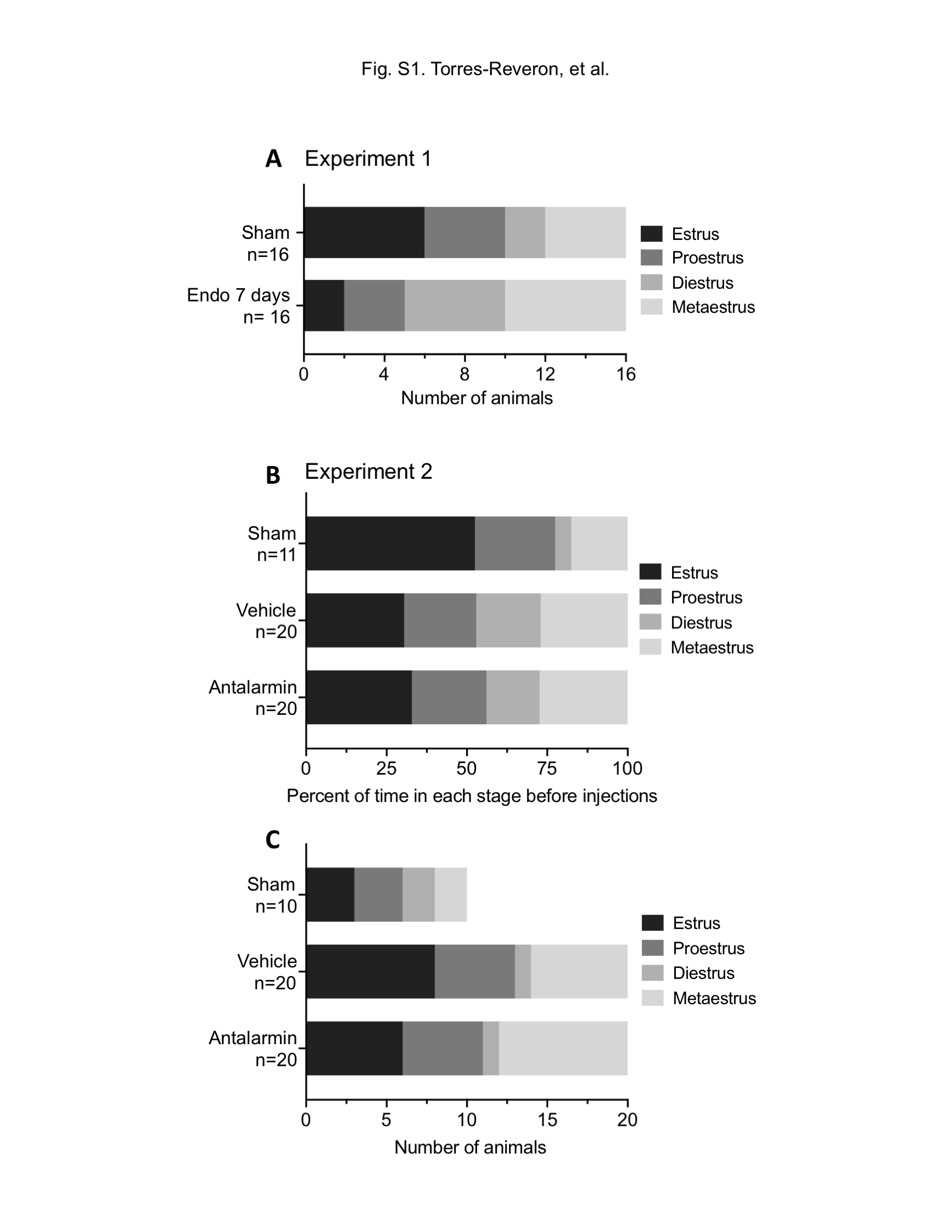

Supplement: S1 Fig — (A) The number of rats that were at each stage of the estrous cycle the day of behavioral testing and sacrifice for experiment 1. Estrous cycle before endometriosis surgery was not assessed in these rats. (B) Estrous cycle in rats for experiment 2 was assessed for 10 days prior to induction surgery. The percent of time that each experimental group spent in each of the phases of the estrous cycle. (C) The number of rats that were on each phase of the estrous cycle on the day of behavioral testing and sacrifice, 60 days after the induction surgery. Note that one rat in the sham group had irregular cycles and was removed from the group. (TIF) [file pone.0197698.s001.tif]

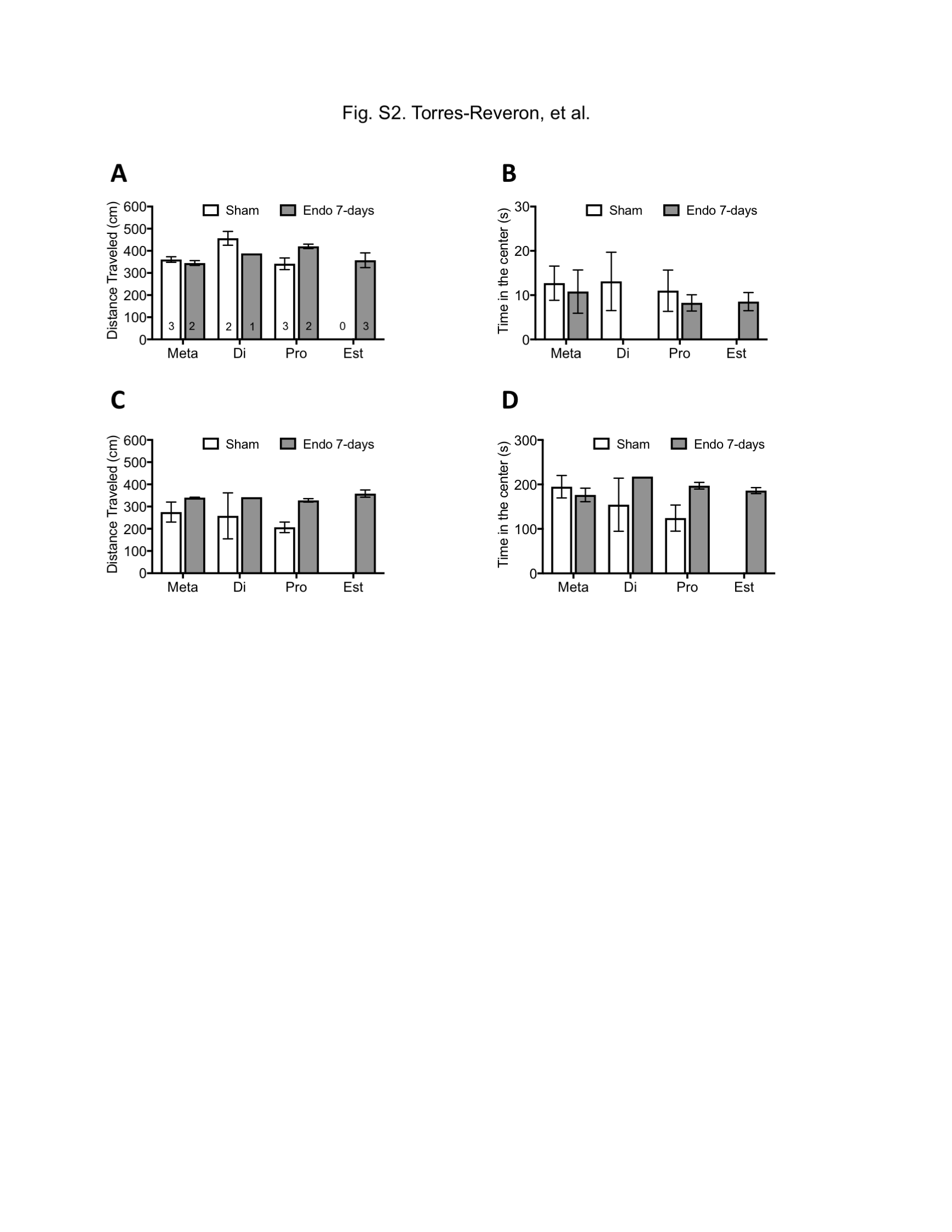

Supplement: S2 Fig — Estrous cycle stage at the time of sacrifice was measured by vaginal smear. We did not pre-planned to sacrifice at any particular stage of the cycle but rather at exactly 7 days after the endometriosis induction. (A and B) Total distance traveled in the open field and time spent in the center. (C and D) Total distance traveled in the zero maze and time spent in the open arms of the maze. Parametric analyses were not performed due to the small number of animals in each estrous cycle group. Numbers at the bottom of bars in panel A represent the number of animals per group per stage of estrous cycle. Bars represent mean ± S.E.M. in this and all subsequent supplemental figures. (TIF) [file pone.0197698.s002.tif]

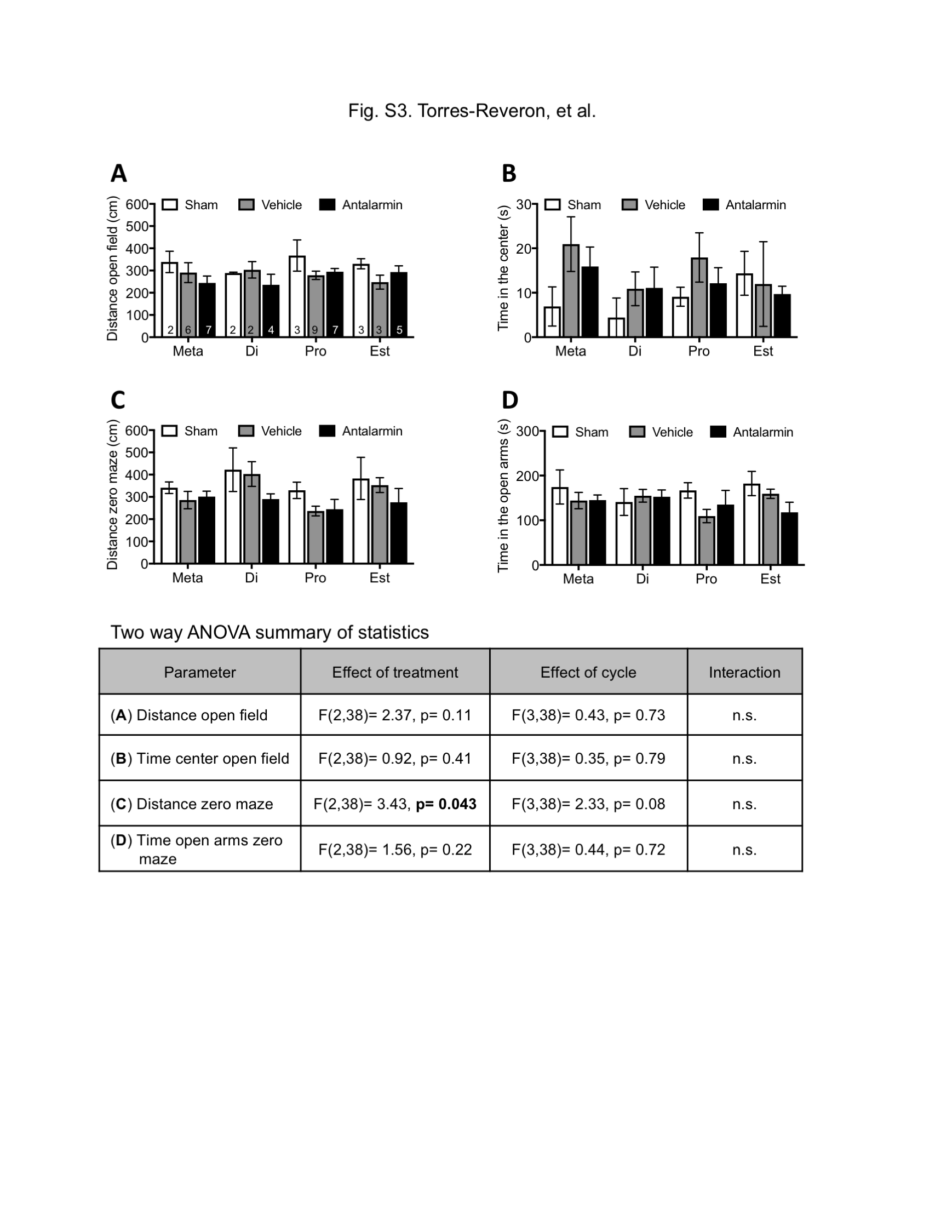

Supplement: S3 Fig — Similar to experiment 1, estrous cycle stage at the time of sacrifice was measured by vaginal smear. We did not pre-planned to sacrifice at any particular stage of the cycle but rather at 60 days after the endometriosis induction. (A and B) Total distance traveled in the open field and time spent in the center. (C and D) Total distance traveled in the zero maze and time spent in the open arms of the maze. The table illustrates the statistical results of the Two-way ANOVAs for each panel showing no effects of estrous cycle. Numbers at the bottom of bats in panel A represent the number of animals per group per stage of estrous cycle. It should be noted that the number of animals per group in the diestrus stage of the estrous cycle was small. (TIF) [file pone.0197698.s003.tif]

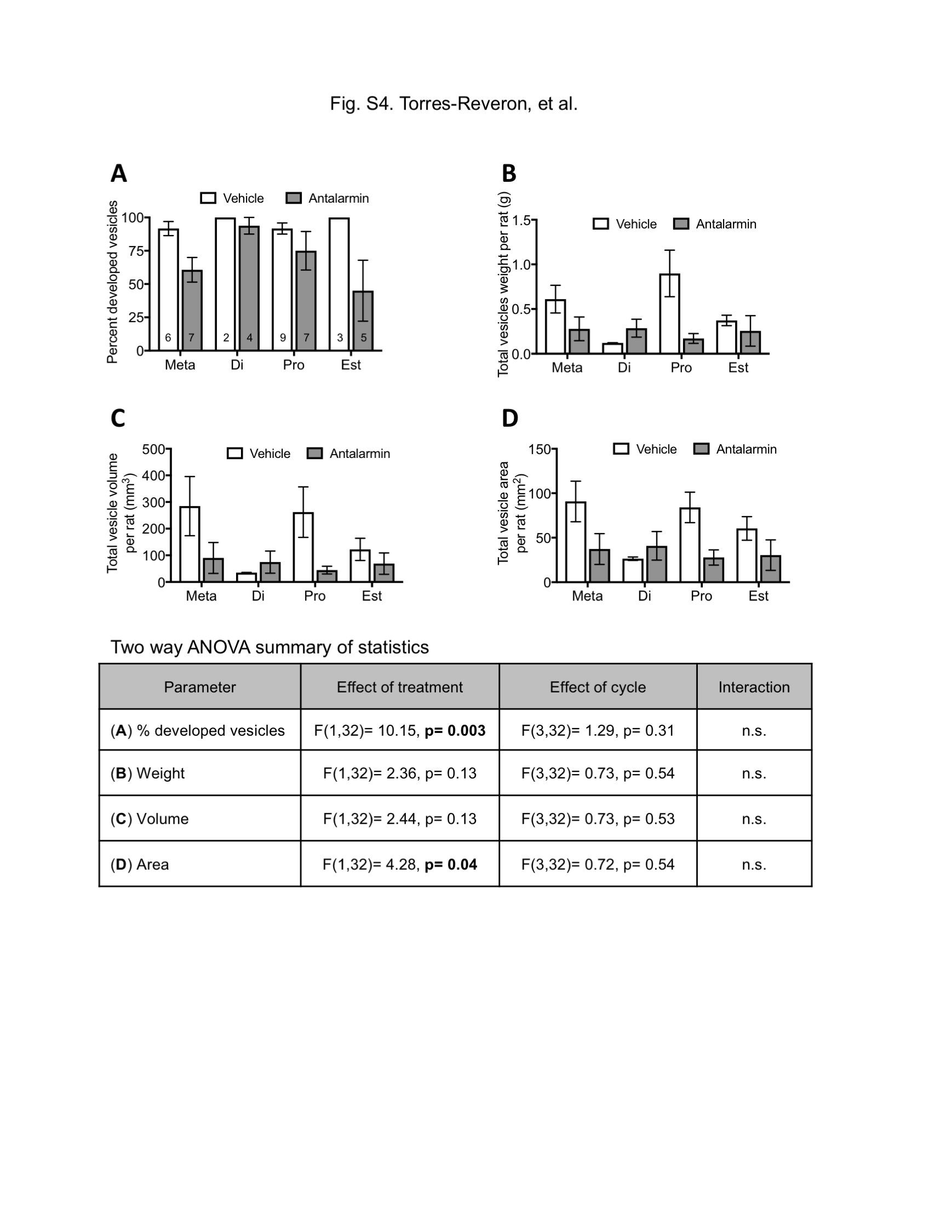

Supplement: S4 Fig — (A) Percent developed vesicles. (B) Group means of the total vesicle weight per rat. (C) Group means for the total vesicle volume per rat. (D) Group means for the total vesicle area per rat. The table illustrates the statistical results of the Two-way ANOVAs for each panel showing no effects of estrous cycle. No interactions of estrous cycle and treatment were observed. Numbers at the bottom of bars in panel A represent the number of animals per group, per stage of estrous cycle. (TIF) [file pone.0197698.s004.tif]
